# Probing the Interaction of a Therapeutic Flavonoid, Pinostrobin with Human Serum Albumin: Multiple Spectroscopic and Molecular Modeling Investigations

**DOI:** 10.1371/journal.pone.0076067

**Published:** 2013-10-08

**Authors:** Shevin R. Feroz, Saharuddin B. Mohamad, Zarith S. D. Bakri, Sri N. A. Malek, Saad Tayyab

**Affiliations:** 1 Biomolecular Research Group, Biochemistry Programme, Institute of Biological Sciences, Faculty of Science, University of Malaya, Kuala Lumpur, Malaysia; 2 Bioinformatics Programme, Institute of Biological Sciences, Faculty of Science, University of Malaya, Kuala Lumpur, Malaysia; 3 Centre of Research for Computational Sciences and Informatics for Biology, Bioindustry, Environment, Agriculture and Healthcare, Institute of Biological Sciences, Faculty of Science, University of Malaya, Kuala Lumpur, Malaysia; Russian Academy of Sciences, Institute for Biological Instrumentation, Russian Federation

## Abstract

Interaction of a pharmacologically important flavonoid, pinostrobin (PS) with the major transport protein of human blood circulation, human serum albumin (HSA) has been examined using a multitude of spectroscopic techniques and molecular docking studies. Analysis of the fluorescence quenching data showed a moderate binding affinity (1.03 × 10^5^ M^−1^ at 25°C) between PS and HSA with a 1∶1 stoichiometry. Thermodynamic analysis of the binding data (Δ*S* = +44.06 J mol^−1^ K^−1^ and Δ*H* = −15.48 kJ mol^−1^) and molecular simulation results suggested the involvement of hydrophobic and van der Waals forces, as well as hydrogen bonding in the complex formation. Both secondary and tertiary structural perturbations in HSA were observed upon PS binding, as revealed by intrinsic, synchronous, and three-dimensional fluorescence results. Far-UV circular dichroism data revealed increased thermal stability of the protein upon complexation with PS. Competitive drug displacement results suggested the binding site of PS on HSA as Sudlow’s site I, located at subdomain IIA, and was well supported by the molecular modelling data.

## Introduction

Dietary flavonoids form an important class of phytonutrients available from plant sources. These phenolic compounds offer numerous health benefits to humans owing to their antioxidant, antiinflammatory, anticarcinogenic, immunostimulating, and antimicrobial activities [1], [2]. Thus, screening of flavonoids for the development of potential drugs, dietary supplements, and functional food products has become a research trend in recent years [3].

**Figure 1:**
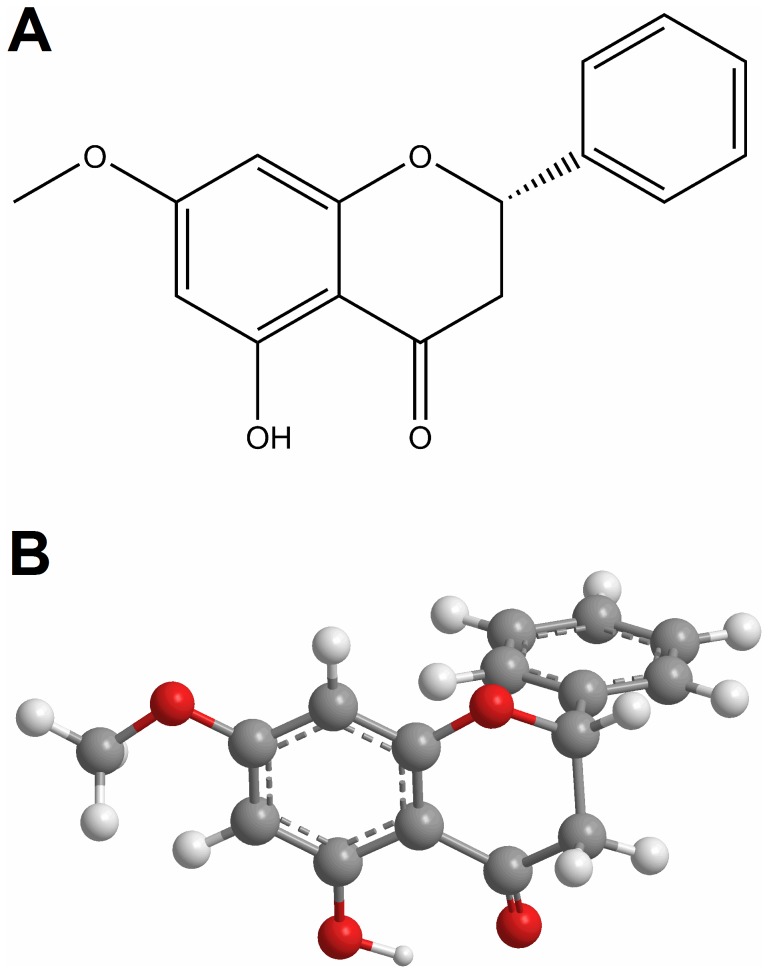
Structural representation of pinostrobin. (A) Chemical structure and (B) ball-and-stick model.

Pinostrobin (PS) (Figure 1), a member of flavanone family of flavonoids has been shown to possess many therapeutic activities. Whereas anticancer activity of PS has been demonstrated in human breast cancer cell lines [2], inhibition of the replication of herpes simplex virus-1 and its inactivation observed in presence of PS has reflected its antiviral activity [4]. PS has also been identified as an inducer of mammalian phase II detoxication enzyme [1]. In addition, the ability of PS to inhibit the cyclooxygenase enzyme pathway has revealed its potential as an antiinflammatory agent [5].

In the human body, the bioavailability, distribution and metabolism of many bioactive compounds depend on their association with plasma proteins [6] and this interaction directly affects their pharmacokinetic and pharmacodynamic properties. For instance, protein binding of a bioactive compound leads to improved solubility in plasma, reduced toxicity, protection against oxidation, as well as prolonged *in vivo* half-life of the bound molecule [6], [7]. On the other hand, such interaction may also result in structural alterations in the protein, thus affecting its functional properties [6]. Hence, the study of ligand–protein interaction is of fundamental importance in unraveling the nature of their action in the body.

Being the major transport protein of the mammalian blood circulation, human serum albumin (HSA), binds a number of endogenous and exogenous compounds. The single polypeptide chain of 585 amino acid residues, comprised of six subdomains, namely, IA, IB, IIA, IIB, IIIA and IIIB is organized into a heart-shaped molecule [6], [7]. Many molecules have been found to bind with high affinity to either one of the two major binding sites on HSA, designated as site I and II [6]–[8]. X-ray crystallographic studies on HSA have later mapped sites I and II to specialized cavities centered in subdomains IIA and IIIA, respectively [9].

Despite the publication of many reports illustrating the various pharmacological properties of PS [1], [2], [4], [5], a comprehensive study on its interaction with the major transport protein of the human blood circulation is yet to be presented. In an attempt to achieve a better understanding of the transport of PS in human circulation, we report here in detail the binding characteristics of the PS–HSA interaction as investigated by multiple spectroscopic probes. In addition, competitive displacement experiments and molecular modeling studies have also been performed to reveal the location of the PS binding site on HSA as well as the forces involved in the binding reaction.

## Materials and Methods

### Materials

Essentially fatty acid free HSA, warfarin (WFN), bilirubin (BR) and ketoprofen (KTN) were procured from Sigma Chemical Co. (St. Louis, MO). Diazepam (DZM) was a product of Lipomed AG (Arlesheim, Switzerland). Silica gel 60 was purchased from Merck KGaA (Darmstadt, Germany). PS was purified in our laboratory following procedures described below. All other chemicals used were of analytical grade.

### Isolation and Purification of PS

Fresh rhizomes (6000 g) of the plant *Boesenbergia rotunda* were dried, ground to fine powder (430 g) and soaked twice in 90% (v/v) methanol. The extracting solvent was evaporated under vacuum on a rotary evaporator to obtain crude methanolic extract (40 g). The extract was treated with hexane to obtain the hexane-insoluble residue (33 g) which was extracted further with chloroform. The chloroform-soluble extract (5.0 g) was subjected to vacuum liquid column chromatography on a silica gel 60 column (0.063 × 0.200 m). Elution was initially performed with hexane followed by the gradual increase in the solvent polarity using increasing volumes of chloroform/acetone mixture. Fraction 1 of the total nine fractions, thus obtained, was subjected to repeated recrystallization to obtain the pure compound (13.7 mg). The compound was analyzed using GCMS and NMR spectroscopy and was identified as PS.

### Preparation of Protein and Ligand Solutions

HSA stock solution was prepared in 10 mM Tris-HCl buffer, pH 7.4 and its concentration was determined spectrophotometrically using a specific extinction coefficient of 5.3 at 280 nm [10]. Stock solution of WFN was made by dissolution in methanol and its concentration was determined using a molar extinction coefficient of 13,610 at 310 nm [11]. BR stock solution was prepared by dissolution in 0.5 M NaOH containing 1 mM EDTA [12], and diluting it with the above buffer. Its concentration was measured spectrophotometrically using a molar extinction coefficient of 47,500 at 440 nm [13]. The BR solution was prepared fresh and used within 2 h. All procedures involving BR were performed under minimal light to avoid its photodegradation. Stock solutions of KTN, DZM and PS were prepared by dissolving known amounts of their crystals in appropriate volumes of ethanol. Working solutions of the above ligands were prepared from their stock solutions after dilution with 10 mM Tris-HCl buffer, pH 7.4.

### Fluorescence Spectroscopy

Fluorescence measurements were carried out on a Jasco FP-6500 spectrofluorometer using a 1 cm path length quartz cuvette placed in a thermostatically controlled water-jacketed cell holder. The emission spectra of the HSA solutions (3 µM) in the absence and presence of PS (0–22.5 µM with 1.5 µM intervals) were recorded in the wavelength range of 300–380 nm upon excitation at 280 nm.

Synchronous fluorescence measurements were performed by scanning the protein samples in the wavelength range, 280–320 nm and 310–370 nm while keeping the difference between excitation and emission wavelengths (Δλ) of 15 and 60 nm, respectively. The concentration of HSA was fixed at 3 µM while the PS concentration was varied in the range, 0–22.5 µM with 1.5 µM intervals.

The 3-D fluorescence spectra of HSA (3 µM) both in the absence and presence of 9 µM PS were acquired by recording the emission spectra in the wavelength range, 220–500 nm while setting the excitation wavelength in the range, 220–350 nm with 10 nm intervals.

### Circular Dichroism (CD) Spectroscopy

CD measurements were performed on a Jasco J-815 spectropolarimeter equipped with a Jasco PTC-423S/15 temperature controller. Measurements were made under constant nitrogen flow using quartz cuvettes of 1 mm and 10 mm path length in the far-UV and near-UV regions, respectively.

For thermal stability studies, ellipticity values of HSA solutions (3 µM) both in the absence and the presence of 15 µM PS were recorded at 222 nm in the temperature ranges, 25–80°C and 25–100°C in the forward and reverse directions. The PS–HSA mixture was incubated for 1 h at room temperature to achieve equilibrium prior to CD measurements. The samples were incubated for 3 min each temperature before the ellipticity values were recorded. Transformation of CD values into mean residue ellipticity (MRE) was performed as described earlier [14].

### PS–HSA Interaction Studies

Binding of PS to HSA was studied fluorometrically at four different temperatures, i.e., 15, 25, 35 and 45°C. Titration experiments involving HSA and PS were performed following the method described earlier [15]. Due to significant absorption of PS near the excitation wavelength, the fluorescence data were corrected for the inner filter effect in the same way as described by Lakowicz [16] using the following equation:
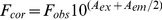
(1)where *F_cor_* is the corrected fluorescence, while *F_obs_* is the measured fluorescence. *A_ex_* and *A_em_* represent the difference in the absorbance values of the protein, observed upon addition of the ligand at excitation (280 nm) and emission wavelengths (300−380 nm), respectively.

In order to verify the mechanism of quenching, the fluorescence data were treated according to the Stern-Volmer equation [16]:
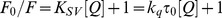
(2)where *F_0_* and *F* are the fluorescence intensities in the absence and presence of the quencher, respectively, *K_SV_* is the Stern-Volmer constant and [*Q*] is the quencher concentration [16]. To calculate the value of the bimolecular quenching constant, *k_q_*, the value of the HSA fluorescence lifetime in the absence of quencher (τ_0_) was taken as 6.38 × 10^−9^ s [17].

The association constant, *K_a_* as well as the stoichiometry of binding, *n* for PS–HSA interaction were determined following the method described earlier [18] which does not take into account any assumptions for the ligand concentration as used in the modified Stern-Volmer equation and the double logarithmic plot of log (*F_0_−F*)/*F* against log [*Q*] [19]. The method involves the use of the following equation:
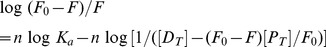
(3)where [*D_T_*] and [*P_T_*] refer to the total concentration of the ligand and the protein, respectively. *F_0_* and *F* have the same significance as described above.

Analysis of thermodynamic parameters of PS–HSA interaction was made using the van’t Hoff equation:
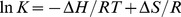
(4)where *K* is the binding constant, Δ*H* is the enthalpy change, *R* is the gas constant, *T* is the absolute temperature and Δ*S* is the entropy change. Values of Δ*H* and Δ*S*, thus calculated using the above method were assumed to be constant at all the four temperatures studied. Free energy change (Δ*G*) of the binding reaction was determined using the following relationship after substituting the values of Δ*H* and Δ*S* as obtained above.
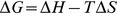
(5)


### Determination of Binding Distance

According to Förster’s energy transfer theory, the distance between the donor (Trp-214 of HSA) and acceptor (PS), *r* as well as the efficiency of the transfer of energy, *E* can be calculated from the following equation [16]:
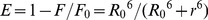
(6)where *F* and *F_0_* are the fluorescence intensities of HSA in the presence and absence of PS, and *R_0_* is the Förster critical distance at which the efficiency of the energy transfer is at 50% and is described by the following relationship [16]:
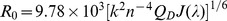
(7)where k^2^ is the spatial factor of orientation of the transition dipoles, *n* is the refractive index of the medium, Q_D_ is the fluorescence quantum yield of the donor in the absence of acceptor and *J* is the integral overlap between the donor emission and acceptor absorption. By taking the values of k^2^ as 2/3 (for the random averaging of the donor-acceptor pair dipoles [16]), *n* as 1.336 (for the refractive index of dilute aqueous mediums [20]), and Q_D_ as 0.118 (for the fluorescence quantum yield of native HSA [16]; *J* and R_0_ were calculated with the help of LabVIEW 8.2 software (National Instruments Corp., Austin, TX). Although the donor-acceptor transition dipoles in PS–HSA complex are not strictly free in terms of orientation, even modest reorientations of the dipoles might have not produced any significant alteration to the value of k^2^ as 2/3 [21].

### Characterization of the PS Binding Site

Displacement of BR by PS was studied by monitoring the effect of the addition of PS at increasing concentrations (0–80 µM with 10 µM intervals) on the visible CD spectra of BR–HSA (1∶1) complex (10 µM each) in the wavelength range of 300–500 nm.

The displacing effect of PS on the binding of WFN to HSA was investigated by recording the fluorescence spectra of WFN–HSA (1∶1) complex (3 µM each) in the wavelength range of 360–480 nm upon exciting the complex at 335 nm, both in the absence and presence of increasing PS concentrations (0–24 µM with 3 µM intervals).

For competitive binding experiments involving DZM, the CD spectra of the DZM–HSA complex (20 µM DZM +10 µM HSA) were recorded in the wavelength range of 250–350 nm both in the absence and presence of increasing PS concentrations (0–80 µM with 10 µM intervals).

The influence of PS on the KTN–HSA complex (20 µM KTN +10 µM HSA) was probed by recording the CD spectra of the complex in the wavelength range of 300–400 nm both in the absence and presence of increasing PS concentrations (0–80 µM with 10 µM intervals).

Site marker–HSA mixtures were preincubated for 1 h before the addition of PS, followed by another 1 h of incubation with PS prior to spectral measurements. For experiments involving BR, both incubation times were set to 15 min. All procedures were carried out at 25°C.

### Docking Studies

Molecular docking, visualization and rendering simulation were performed using AutoDock 4.2 [22] and AutoDockTools 1.5.4 (ADT) [23]. The structure of PS was drawn using ACD/ChemSketch Freeware (Advanced Chemistry Development Inc. Ontario, Canada), 3-D optimized and exported as a mol file. The geometry optimization of PS was refined with the VegaZZ 2.08 [24] batch processing MOPAC script (mopac.r; keywords: MMOK, PRECISE, GEO-OK) using AM1 semiempirical theory [25] and then converted and stored as a mol2 file. For the docking study, the PS non-polar hydrogens were merged and rotatable bonds were defined. Three crystal structures of HSA (PDB IDs: 1BM0, 2.5 Å resolution; 2BXD, 3.05 Å and 2BXF, 2.95 Å) were downloaded from the Protein Data Bank for the docking analysis [26]. All water and ligand molecules were removed from the structures, and the atomic coordinates of chain A of these crystal structures were stored in a separate file and used as input for ADT. Polar hydrogens were added to the protein structure and Kollman united atom partial charges were assigned. During the docking process, the protein was kept rigid, while all the torsional bonds of ligand were set free. The ligand binding site of the protein was defined by a 70 × 70 × 70 grid point with a grid space of 0.375 Å. At subdomain IIA, the grid boxes were centered at x = 35.26, y = 32.41 and z = 36.46 for 1BM0; (5.101, −13.346, 7.444) for 2BXD and (1.333, −10.093, 8.189) for 2BXF. On the other hand, for subdomain IIIA, the grid boxes were centered at (14.42, 23.55, 23.21), (15.226, 4.383, −7.693) and (5.276, 4.635, −10.078), for 1BM0, 2BXD and 2BXF, respectively. Lamarckian genetic algorithm with local search was used as the search engine, with a total of 100 runs for each binding site. In each run, a population of 150 individuals with 27 000 generations and 250 000 energy evaluations were employed. Operator weights for crossover, mutation and elitism were set to 0.8, 0.02, and 1, respectively. For local search, default parameters were used. Root-mean-square-deviation of 2.0 Å was used as a criterion for cluster analysis of the docking results. The protein–ligand complex was visualized and analyzed using ADT.

## Results and Discussion

### Fluorescence Quenching Mechanism and Binding Characteristics of PS–HSA Interaction

Investigations into binding of a ligand to a protein often involve quenching of the protein fluorescence as a result of its interaction with a particular ligand. The phenomenon of fluorescence quenching can be attributed to several molecular mechanisms including excited-state reactions, molecular rearrangements, energy transfer, ground-state complex formation, and collisional quenching [16].

**Figure 2:**
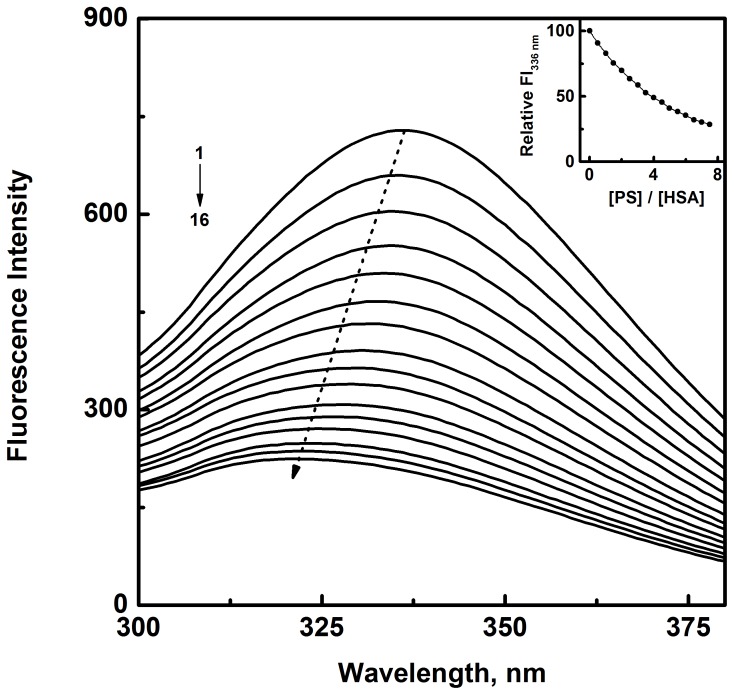
Fluorescence quench titration of HSA with increasing PS concentrations. [HSA] = 3 µM, [PS] = 0–22.5 µM with 1.5 µM intervals (1–16), λ_ex_ = 280 nm studied in 10 mM Tris-HCl buffer, pH 7.4, 25°C. Arrow depicts the blue shift in the emission maximum of HSA with increasing PS concentrations. Inset shows the decrease in the relative fluorescence intensity of HSA at 336 nm (FI_336 nm_) with increasing PS/HSA molar ratios.

As seen from Figure 2, HSA exhibited an emission spectrum in the wavelength range, 300–380 nm with an emission maximum at 336 nm upon excitation at 280 nm, a characteristic of proteins containing Trp residues [16]. Addition of increasing PS concentrations to HSA solution produced a progressive decrease in the fluorescence intensity and significant blue shift in the emission maximum, suggesting the binding of PS to HSA. About 72% decrease in the fluorescence intensity at 336 nm (inset of Figure 2) and 14 nm blue shift were observed at the highest PS concentration used in this study. Whereas fluorescence intensity is influenced by change in the polarity of the microenvironment as well as movement of charged groups in the vicinity of fluorophores, hydrophobic changes in the microenvironment primarily dictate the shift in the emission maximum [27]. Hence, shift in the emission maximum is a better index to evaluate any alteration in the hydrophobicity of the binding region. Therefore, fluorescence characteristics exhibited by HSA upon addition of PS are highly suggestive of conformational change in the protein representing transfer of the fluorophore(s) to a more hydrophobic environment, thus confirming the binding of PS to HSA.

**Figure 3:**
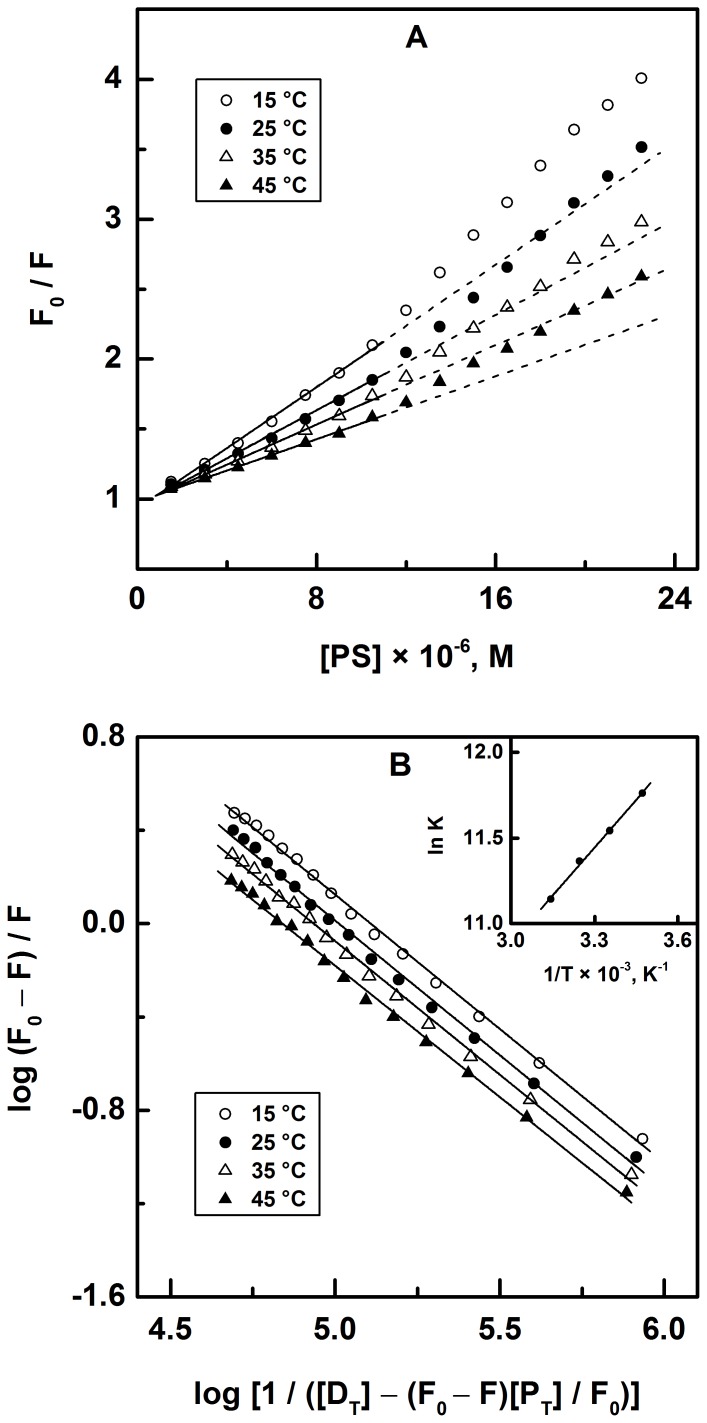
Analysis of fluorescence quenching data. (A) Stern–Volmer and (B) 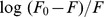
against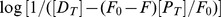 plots of PS–HSA system at different temperatures. Inset of (B) shows the van’t Hoff plot for PS–HSA interaction.

Quenching of protein fluorescence can be classified as either a dynamic or a static process, which can be distinguished based on their temperature dependence. Higher temperatures result in faster diffusion, hence the larger amounts of collisional quenching. Conversely, higher temperatures lead to the dissociation of non-covalently bound complexes resulting in a decrease in static quenching [16]. In order to validate the mode of quenching, titration experiments of HSA with PS were performed at four different temperatures, i.e., 15, 25, 35 and 45°C and the data were analyzed according to Eq. 2. The Stern-Volmer plots (Figure 3A) obtained at these temperatures showed linearity in the lower PS concentration zone and produced an upward curvature at higher PS concentrations. Therefore, only the linear zones of Stern-Volmer plots were selected for regression analysis in order to determine the value of the Stern-Volmer constants, *K_SV_* as listed in Table 1. A progressive decrease in the *K_SV_* values with increasing temperature clearly suggested that the static quenching mechanism involving the formation of a non-covalent PS–HSA binary complex was followed. This was supported by the calculation of the bimolecular quenching constant, *k_q_* values from the *K_SV_* values which were found to fall in the range of 1.71–0.88 × 10^13^ M^−1 ^s^−1^ at all the temperatures studied. Values of *k_q_* higher than the diffusion-controlled limit (∼10^10^ M^−1 ^s^−1^) indicated complex formation between PS and HSA [16].

**Table 1:** Binding and thermodynamic parameters for the interaction between PS and HSA, studied at different temperatures, pH

*T*, °C	*K_SV_*, M^−1^	*K_a_*, M^−1^	*n*	Δ*S*, J mol^−1^ K^−1^	Δ*H*, kJ mol^−1^	Δ*G*, kJ mol^−1^
15	1.09×10^5^	1.28×10^5^	1.15			−28.17
25	8.60×10^4^	1.03×10^5^	1.15	+44.06	−15.48	−28.61
35	7.15×10^4^	8.57×10^4^	1.15			−29.05
45	5.60×10^4^	6.92×10^4^	1.13			−29.49

Values of the association constant, *K_a_* and the stoichiometry of the binding, *n* for PS–HSA interaction at different temperatures were obtained from the logarithmic plots, shown in Figure 3B and the values, thus obtained, are listed in Table 1. The values of *K_a_* indicate intermediate affinity between PS and HSA and were found similar to the values obtained for the interaction of other phytochemicals with serum albumins [3], [15], [21]. As anticipated, the *K_a_* values showed inverse correlation with temperature since the forces involved in the complex formation would have been weakened with increasing temperature. *K_a_* values in the range 1–15 × 10^4^ M^−1^ have been reported for a large number of ligand binding studies to albumin [6], [7]. Such intermediate value of the binding constant seems appropriate for the transport of PS in the blood circulation and its disassociation at the target site, as a large value of *K_a_* would prevent the release of the ligand from the protein, thus hindering its action in the body [6]. The number of binding sites for PS on the HSA molecule was found to be around 1.1 at all temperatures studied; indicating a 1∶1 binding stoichiometry.

### Energetics of PS–HSA Interaction and Binding Forces

Quantitative evaluation of the energetics of protein-ligand interaction is crucial as it provides valuable information regarding binding forces. Hence, thermodynamic parameters of PS–HSA interaction were determined from the van’t Hoff plot (inset of Figure 3B) using *K_a_* values obtained at different temperatures. Values of Δ*H* and Δ*S* as obtained from the van’t Hoff plot, as well as that of Δ*G* obtained at four different temperatures using Eq. 5, are listed in Table 1. The favorable entropic contribution as reflected from the positive Δ*S* value obtained for PS–HSA interaction can be attributed to several phenomena including hydrophobic interactions, and desolvation of the binding site, which involves the destruction of the ordered solvent layers surrounding the ligand and the protein binding site and removal of solvent molecules from the binding pocket [28], [29]. The role of hydrophobic interactions in the PS–HSA complexation can be rationalized from the structural features of PS, which possesses two benzene rings connected by a tetrahydropyran ring system giving it a non-polar character. The involvement of ionic forces in the binding reaction between PS and HSA is highly improbable due to a significantly higher value of Δ*H* obtained for this reaction as these forces are characterized by a Δ*H* ≈ 0 [30]. In addition, absence of any ionizable group in PS further rules out the participation of ionic forces in PS–HSA interaction. A negative Δ*H* value obtained for PS–HSA system can account for the involvement of hydrogen bonding and/or van der Waals forces [29], [30]. This was further confirmed by docking experiments which predicted the details of the hydrogen bonds likely to occur between the functional groups of PS and amino acid residues of the protein in the vicinity of the binding site as discussed in later. Taking into consideration the possibility of several short-range interactions in PS–HSA complexation, it would be inconceivable to assume the involvement of a single acting force in PS–HSA interaction. Therefore, hydrophobic and van der Waals forces along with hydrogen bonds are believed to contribute collectively to the overall energetics of PS–HSA interaction.

### Intermolecular Binding Proximity between PS and HSA

The phenomenon of fluorescence resonance energy transfer (FRET) takes place between a donor in the excited state and an acceptor due to dipole–dipole interactions between the molecules [16]. The extent of this phenomenon depends on the magnitude of overlapping between donor emission and acceptor absorption spectra, donor quantum yield, orientation of the donor and acceptor transition dipoles, and the distance between the donor and acceptor molecules [16]. Being distance specific, FRET can be used to determine the distance between the donor and acceptor molecules.

**Figure 4:**
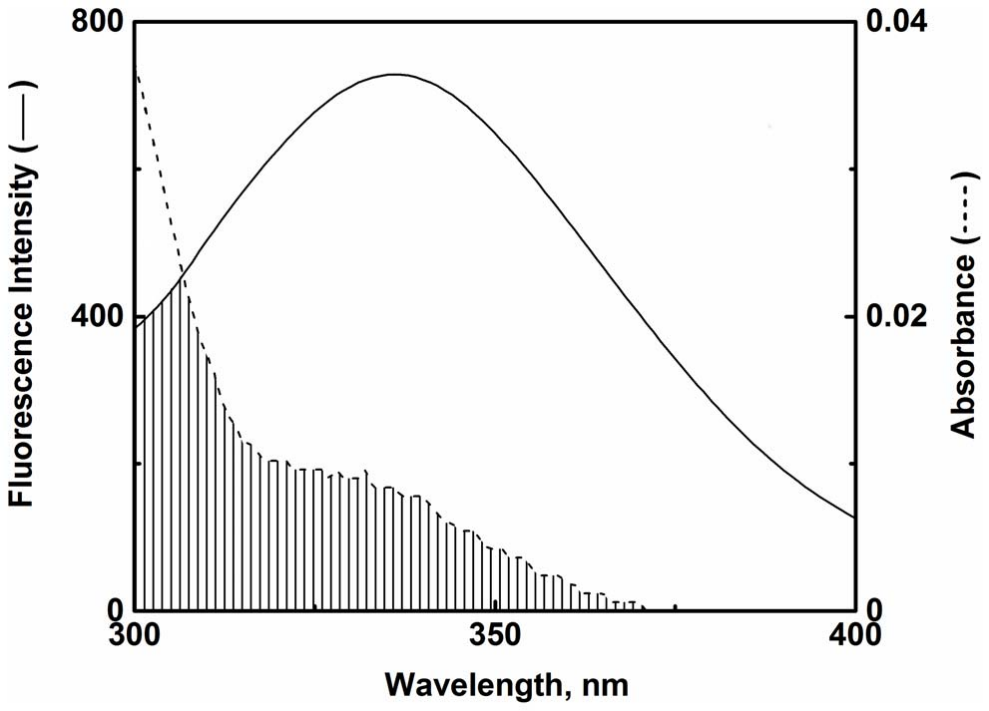
Spectral overlap between the fluorescence spectrum of HSA and absorption spectrum of PS. Both spectra were recorded at 25°C in 10 mM Tris-HCl buffer, pH 7.4. The concentrations of HSA and PS were 3 µM each.

A significant overlap between the emission spectrum of HSA and PS absorption spectrum was observed (Figure 4), suggesting possibility of energy transfer between these molecules. As described in the ‘Materials and Methods’ section, values for *J*, *E*, *R_0_* and *r* were calculated as 2.814 ×10^−15^ M^−1^ cm^3^, 0.214, 1.983 nm and 2.46 nm, respectively. The reliability of *R_0_* and *r* values calculation was evident from the satisfying criteria of 0.5 *R_0_*< *r* <2 *R_0_* [16]. Furthermore, the value of *r* was within the range, 2–8 nm indicating high probability of energy transfer between PS and HSA [31]. Additionally, the larger value of *r* compared to *R_0_* also supported the static quenching mechanism observed for PS–HSA interaction [32]. It is important to note that the above value of *r* was obtained by substituting the value of *k^2^* as 2/3 in Eq. 7, which assumes random free rotation of the transition dipoles. Hence, it might not accurately reflect the arrangement of the donor-acceptor pairs in our system. Nevertheless, due to the sixth power dependence of *r* on *k^2^*, the error in the calculation of *r* can be no larger than 35% compared to values of *r* calculated using other possible value of *k^2^* [16].

### PS-Induced Conformational Alterations in HSA

#### Synchronous fluorescence

**Figure 5:**
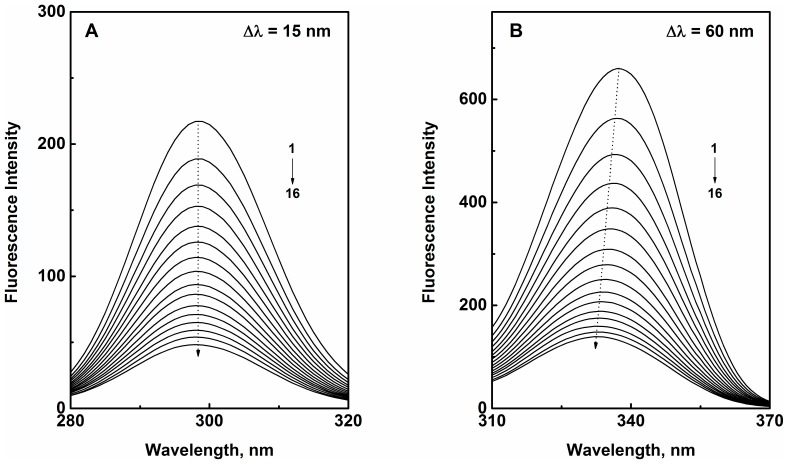
Synchronous fluorescence spectra of HSA obtained in the absence and presence of increasing PS concentrations. [HSA] = 3 µM, [PS] = 0–22.5 µM with 1.5 µM intervals (1–16) studied in 10 mM Tris-HCl buffer, pH 7.4, 25°C. The difference between excitation and emission wavelengths (Δλ) was (A) 15 nm and (B) 60 nm. Arrows depict the position of the emission maximum of HSA in presence of increasing PS concentrations.

To gain a better insight of the microenvironmental changes around protein fluorophores upon PS binding, synchronous fluorescence spectra of HSA were recorded in the absence and presence of increasing PS concentrations. If the difference between excitation and emission wavelengths (Δλ) is set to 15 or 60 nm, synchronous fluorescence spectra of the protein shows environmental perturbation around Tyr or Trp residues, respectively [33]. As can be seen from Figure 5A, emission maximum of HSA (298 nm) remained unaltered in the presence of increasing PS concentrations when Δλ = 15 nm; suggesting that the microenvironment around Tyr residues was not significantly affected by the binding reaction. In contrast, a blue shift of 5 nm was observed when Δλ was fixed at 60 nm (Figure 5B), indicating perturbation in the microenvironment around Trp-214 towards a less polar milieu during PS–HSA complexation. Movement of Trp residue to a more non-polar region as well as binding of a non-polar ligand to the binding pocket may account for the observed blue shift in the synchronous fluorescence spectra. Irrespective of the events that led to this phenomenon, it is possible to conclude from these results that the blue shift observed in the fluorescence spectrum of HSA upon PS binding (Figure 2) probably reflects the increased hydrophobicity in the microenvironment around the Trp residue.

#### Three-dimensional (3-D) fluorescence

**Figure 6:**
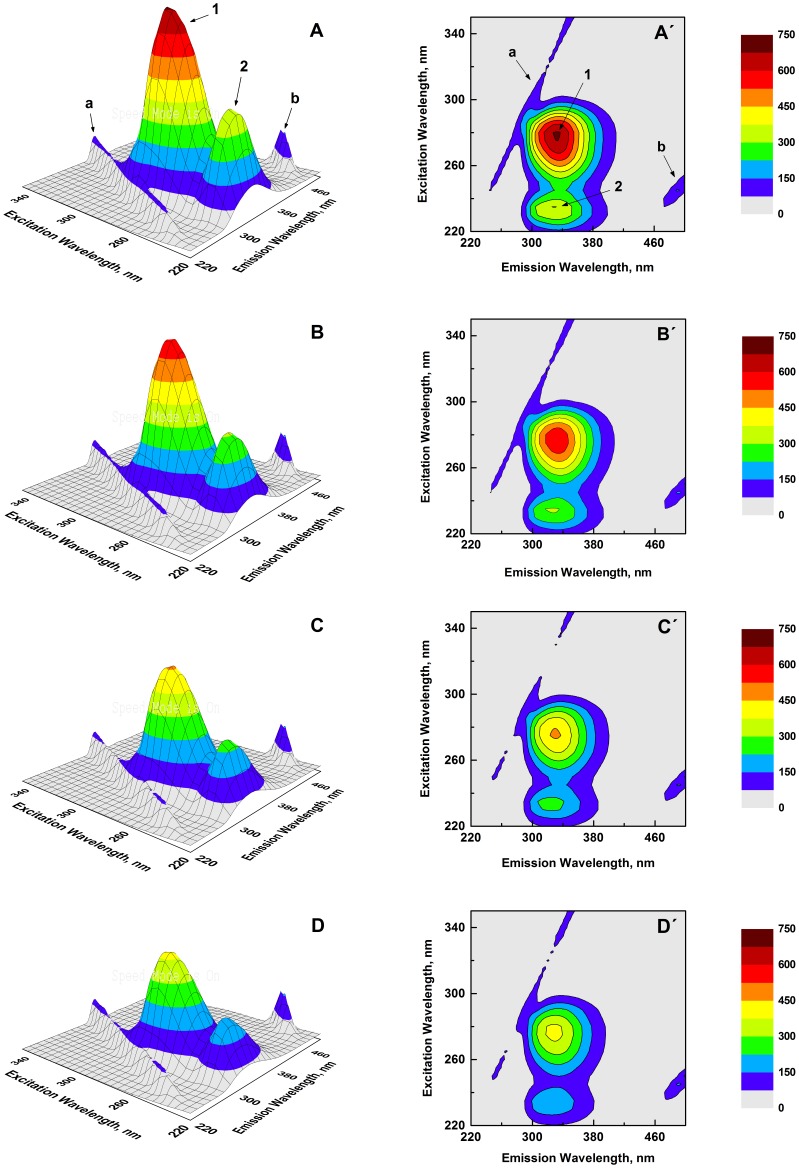
3-D fluorescence spectral projections and corresponding contour maps of HSA and various PS–HSA complexes. (A and A′) Free HSA, (B and B′) 1∶1 PS–HSA, (C and C′) 2∶1 PS–HSA and (D and D′) 3∶1 PS–HSA. The spectra were recorded in 10 mM Tris-HCl buffer, pH 7.4, 25°C using a protein concentration of 3 µM.

In order to further investigate ligand-induced alteration in the protein’s secondary and tertiary structures, 3-D fluorescence spectroscopy of HSA was performed both in the absence and presence of increasing PS concentrations. Figure 6 shows 3-D fluorescence spectra and corresponding contour maps of native HSA (A and A′) and PS–HSA complexes in different molar ratios [1∶1 (B and B′), 2∶1 (C and C′) and 3∶1 (D and D′)]. Spectral characteristics of the 3-D fluorescence spectra in terms of peak position and peak intensity are given in Table 2. Peak ‘a’ (λ_ex_ = λ_em_) and peak ‘b’ (2λ_ex_ = λ_em_) in the spectra refer to the Rayleigh scattering peak and second-order scattering peak, respectively [34]. In addition to these scattering peaks, two other peaks designated as peak ‘1’ (λ_ex = _280 nm) and peak ‘2’ (λ_ex = _235 nm) were also detected. Whereas peak ‘1’ enunciated the fluorescence spectral behavior of Tyr and Trp residues of the protein due to π→π^*^ transition, fluorescence characteristics of the polypeptide backbone conformation as a result of n→π^*^ transition was evident from peak ‘2’ [34]. In view of this, any change in peak ‘1’ and peak ‘2’ characteristics of the protein in the presence of ligand would indicate tertiary and secondary structural alterations, respectively. A clear trend of decreasing fluorescence intensity accompanied by blue shift for both peaks ‘1’ and ‘2’ of HSA was noticed with increasing PS/HSA molar ratios (Table 2); suggestive of significant conformational alteration in HSA involving both secondary and tertiary structures upon specific interaction with PS. These results were in accordance with intrinsic and synchronous fluorescence results, described above.

**Table 2:** Characteristics of three-dimensional fluorescence spectra of native HSA and its complexes with PS at pH 7.4, 25°C.

System	Peak	Peak Position	Intensity
		[λ_ex_/λ_em_, nm/nm]	
HSA	a	230/230→350/350	23.39→96.41
	b	250/500	153.61
	1	280/335	683.13
	2	235/330	359.12
[PS]:[HSA] = 1∶1	a	230/230→350/350	23.02→93.68
	b	250/500	155.66
	1	280/332	580.78
	2	235/326	307.91
[PS]:[HSA] = 2∶1	a	230/230→350/350	22.53→91.79
	b	250/500	153.36
	1	280/330	448.23
	2	235/323	252.40
[PS]:[HSA] = 3∶1	a	230/230→350/350	22.03→92.69
	b	250/500	157.06
	1	280/329	389.85
	2	235/320	219.38

In view of the conformational alterations involving both secondary and tertiary structures as observed above, it seems probable that these conformational changes might have occurred in the vicinity of PS binding site on HSA. This site has been identified as site I based on our drug displacement and molecular docking results. Site I has been shown to lie within the core of subdomain IIA that comprises all 6 helices of the subdomain (residues 177–291) as well as a loop-helix feature (residues 148–154) contributed by subdomain IB [6]. Although the interior of the binding pocket has been characterized as predominantly apolar, two clusters of polar residues; an inner cluster towards the bottom of the pocket (Tyr-150, His-242, Arg-257) and an outer cluster at the entrance (Lys-195, Lys-199, Arg-218, Arg-222) gives a polar character to the binding pocket [35]. Reports on the binding of warfarin, a typical site I ligand, to HSA have suggested that conformational changes in HSA have taken place primarily due to the interaction of these polar clusters with the ligand. This helps drive the relative rotation of domains I and II and has a large impact on one side of site I [35], [36].

### PS-Induced Thermal Stabilization of HSA

**Figure 7:**
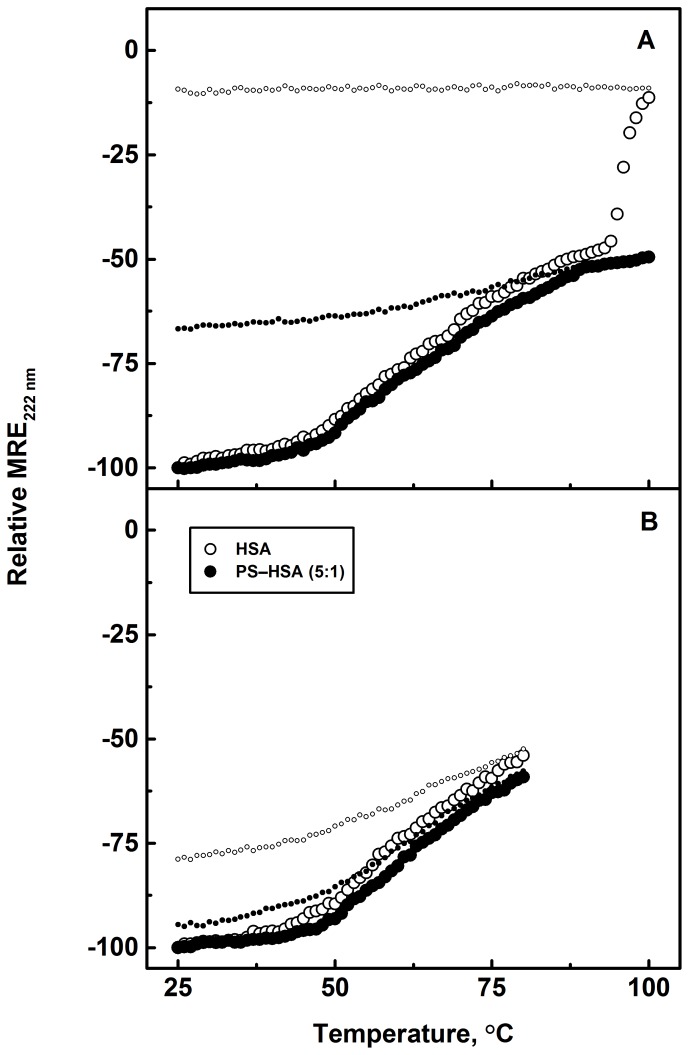
Thermal stability profiles of HSA (3 µM) and 5∶1 PS–HSA complex. MRE values at 222(A) 25–100°C and (B) 25–80°C, obtained in 10 mM Tris-HCl buffer, pH 7.4. The smaller symbols represent the refolding experimental results obtained with the same HSA and PS–HSA systems.

Binding of small molecules to proteins often results in alteration in their thermal stability. Such a change in the protein’s thermal stability has been ascribed to the coupling of binding and unfolding equilibria [37], [38]. In order to investigate the influence of PS–HSA complexation on the thermal stability of HSA, MRE_222 nm_ values of the protein were recorded in the temperature range, 25–100°C and 25–80°C. Renaturation experiments were also performed upon cooling the heated samples down to 25°C to examine the reversibility of the denaturation phenomena. Figure 7A shows thermal denaturation/renaturation profiles of HSA in the absence and presence of 5 molar excess of PS over to HSA in the temperature range, 25–100°C. The denaturation profile of free HSA suggested that the protein’s α-helical structure remained stable up to 45°C and showed significant disruption within the temperature range, 45–87°C, indicating protein denaturation. The sharp drastic decrease in the MRE_222 nm_ value beyond 94°C can be attributed to irreversible unfolding of HSA leading to aggregation, as reflected from the renaturation experiment which showed no change in the MRE_222 nm_ value, and the appearance of visible precipitates in the cuvette. It seems plausible to assume the aggregation of unfolded protein at higher temperature through intermolecular hydrophobic interactions involving exposed hydrophobic patches in the unfolded protein molecules [39]. Presence of PS in the incubation mixture significantly affected the thermal stability of HSA within the temperature range studied. This was evident from the gradual decrease in MRE_222 nm_ at temperatures ≥94°C against the sharp decrease observed in its absence, as well as from the renaturation results where a significant recovery (∼35%) in MRE_222 nm_ value was observed. Furthermore, no sign of precipitation was observed in the renaturation experiments in the presence of PS. Therefore, it is clear that the interaction between PS and HSA stabilized the protein structure against thermal denaturation by reducing the loss in helicity and offering protection against protein aggregation.

Since exposure of the protein to higher temperatures was found to be the cause of irreversible protein denaturation and aggregation, the experiment was repeated within the temperature range, 25–80°C (Figure 7B). Although no significant change in the denaturation profiles was seen when compared to those shown in Figure 7A, remarkable alterations in the renaturation profiles of HSA were noticed. A significant recovery in the native structural characteristics was observed, showing 87% regain in the MRE_222 nm_ value at 5∶1 PS/HSA molar ratio compared to only 55% observed with HSA alone. Hence, these results unequivocally suggested the thermal stabilization of HSA by PS binding.

### Binding Site Specificity of PS on HSA

Several physiological ligands as well as a large number of drugs are known to bind to HSA with high affinity at one of two distinct binding sites namely, site I, located in subdomain IIA and site II, present in subdomain IIIA [6], [7]. In order to investigate if PS exhibits any preference towards these sites, displacement experiments were performed using site marker ligands. The selected markers for site I were BR and WFN; whereas site II was probed using DZM and KTN as the reporter ligands [7].

**Figure 8:**
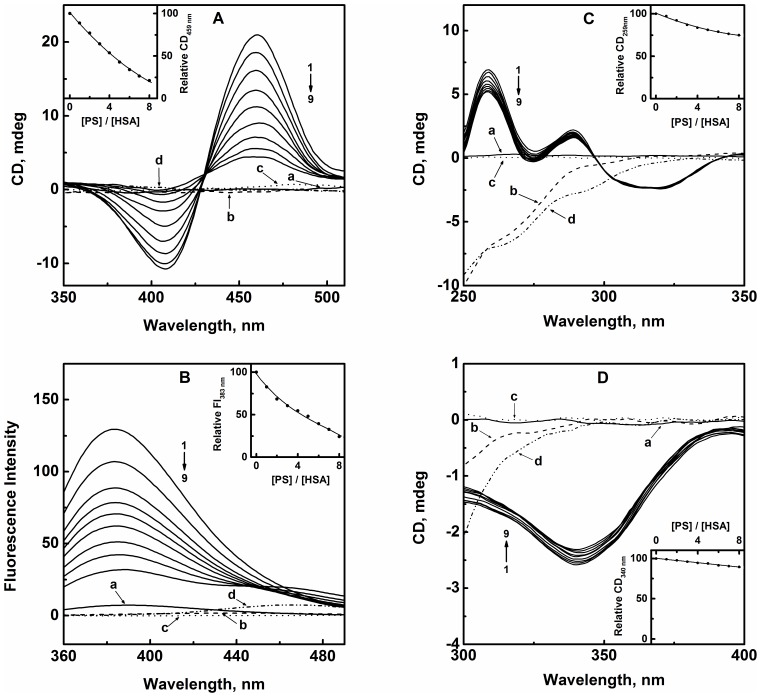
Displacement of site marker ligands bound to HSA in presence of increasing PS concentrations. (A) CD spectra of BR–HSA complex (10 µM each) in the absence (1) and presence (2–9) of increasing PS concentrations (10–80 µM with 10 µM intervals). Spectra marked as ‘a’, ‘b’, ‘c’ and ‘d’ refer to 10 µM BR, 10 µM HSA, 50 µM PS and PS–HSA (5∶1) complex, respectively. Inset shows the decrease in the relative CD value at 459 nm (CD_459 nm_) with increasing PS/HSA molar ratios. (B) Fluorescence spectra of WFN–HSA complex (3 µM each) in the absence (1) and presence (2–9) of increasing PS concentrations (3–24 µM with 3 µM intervals). Spectra labeled as ‘a’, ‘b’, ‘c’ and ‘d’ refer to 3 µM WFN, 3 µM HSA, 15 µM PS and PS–HSA (5∶1) complex, respectively. Inset shows the decrease in the relative fluorescence intensity at 383 nm (FI_383 nm_) with increasing PS/HSA molar ratios. (C) CD spectra of DZM–HSA complex (20 µM DZM +10 µM HSA) in the absence (1) and presence (2–9) of increasing PS concentrations (10–80 µM with 10 µM intervals). Spectra marked as ‘a’, ‘b’, ‘c’ and ‘d’ represent 20 µM DZM, 10 µM HSA, 50 µM PS and PS–HSA (5∶1) complex, respectively. Inset shows the change in the relative CD value at 259 nm (CD_259 nm_) with increasing PS/HSA molar ratios. (D) CD spectra of KTN–HSA complex (20 µM KTN +10 µM HSA) in the absence (1) and presence (2–9) of increasing PS concentrations (10–80 µM with 10 µM intervals). Spectra labeled as ‘a’, ‘b’, ‘c’ and ‘d’ refer to 20 µM KTN, 10 µM HSA, 50 µM PS and PS–HSA (5∶1) complex, respectively. Inset shows the change in the relative CD value at 340 nm (CD_340 nm_) with increasing PS/HSA molar ratios.

Figure 8A shows the effect of increasing PS concentrations on the visible CD spectrum of BR–HSA complex. As can be seen from the figure, BR–HSA complex exhibited a bisignate CD spectrum with a maximum at 459 nm and a minimum at 407 nm. In the absence of any chirality in the free BR molecule (spectrum ‘a’), the above Cotton effect is believed to be the result of the dissymmetry produced in the BR structure upon binding to HSA [40]. Free forms of PS (spectrum ‘c’) and HSA (spectrum ‘b’) as well as their complex (spectrum ‘d’) did not contribute any CD signal in the wavelength range studied; thus, any signal in this wavelength range can be attributed to the complexation between BR and HSA. Addition of increasing PS concentrations to BR–HSA complex led to a significant reduction in the induced CD signal at 459 nm showing around 80% loss at a PS/HSA molar ratio of 8∶1 (inset of Figure 8A). Such a decrease in the CD_459 nm_ signal clearly indicated the displacement of BR from its binding site on HSA in the presence of PS, which was suggestive of the site I of HSA as the preferred binding site for PS.

To substantiate the above findings, another site I marker ligand, WFN was used and the displacing action of PS on WFN–HSA complex was monitored by fluorescence spectroscopy (Figure 8B). WFN in free form produced a weak fluorescence spectrum in the wavelength range, 360–490 nm when excited at 335 nm (spectrum ‘a’). However, a pronounced emission spectrum with an emission maximum at 383 nm was observed upon its complexation with HSA [40]. It is important to note that both free PS (spectrum ‘c’) and HSA (spectrum ‘b’) as well as PS–HSA complex (spectrum ‘d’) did not produce any significant fluorescence spectra in that range. The intensity of the fluorescence spectra of WFN–HSA complex decreased with the addition of PS in a concentration-dependent manner, showing 75% reduction at a PS/HSA molar ratio of 8∶1 (inset of Figure 8B). Decrease in the fluorescence intensity of WFN–HSA complex in the presence of PS suggested the removal of WFN from site I of HSA by PS. These results corroborated the earlier findings obtained with BR displacement experiments.

In order to investigate the possibility of PS binding to site II of HSA, CD spectra of DZM–HSA complex were recorded in the wavelength range, 250–350 nm both in the absence and presence of increasing PS concentrations (Figure 8C). Both free DZM (spectrum ‘a’) and PS (spectrum ‘c’) did not show any significant CD spectra in this wavelength range. However, binding of DZM to HSA produced a CD spectrum, characterized by the presence of two maxima at 259 nm and 289 nm, and a minimum at 319 nm [41]. On the other hand, HSA (spectrum ‘b’) as well as its complex with PS (spectrum ‘d’) produced CD spectra which showed negative CD values throughout the wavelength range studied. The addition of PS to DZM–HSA complex produced little effect on the complexation as reflected from the small change (24%) in the relative CD signal at 259 nm in presence of PS (inset of Figure 8C). This decrease in the CD signal was much smaller compared to the decrease observed with either BR–HSA or WFN–HSA complexes. In view of the negative CD signal at 259 nm shown by PS–HSA complex, one should expect a much larger decrease in the CD_259 nm_ value in the presence of PS, if there had been displacement of DZM from its binding site on HSA by PS. Therefore, a small decrease in the relative CD_259 nm_ value observed with DZM–HSA complex in presence of PS cannot be taken to indicate DZM displacement by PS.

The displacement of KTN, another site II ligand, by PS was also studied using CD spectroscopy in the wavelength range, 300–400 nm. Figure 8D depicts the effect of the addition of increasing PS concentrations on the induced CD spectrum of KTN–HSA complex. As evident from the figure, a negative Cotton effect was induced with a minimum at around 340 nm upon binding of KTN to HSA [41]. On the other hand, both free KTN (spectrum ‘a’) and PS (spectrum ‘c’) exhibited negligible CD signals in the same wavelength range. However, both HSA (spectrum ‘b’) as well its conjugate with PS (spectrum ‘d’) displayed significant negative CD values in the wavelength range, 300–335 nm, beyond which CD signals became insignificant. The CD spectra of KTN–HSA complex showed very slight variation in the presence of increasing PS concentrations. Only 10% decrease in the relative CD_340 nm_ signal was observed at a PS/HSA molar ratio of 8∶1 (inset of Figure 8D). These results suggested that KTN binding to HSA remained largely unaffected in the presence of increasing PS concentrations, thus reflecting differential preferences of these ligands for the two binding sites on HSA. These results were in accordance with the outcome of the above displacement experiments in assigning site I as the preferred binding site of PS on HSA.

### Docking Analysis

**Figure 9:**
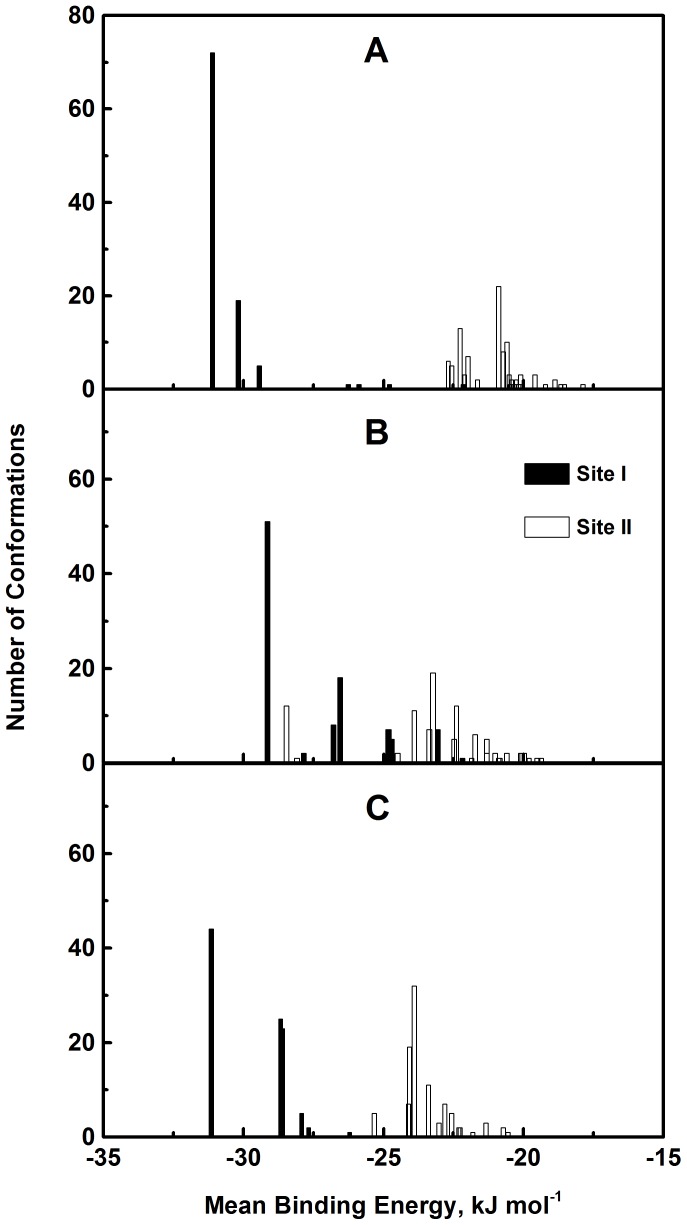
Cluster analysis of PS docking to binding sites I and II of HSA. The different crystal structures of HSA used in the analysis were (A) 1BM0, (B) 2BXD and (C) 2BXF. A total of 100 runs were performed for each binding locus.

A docking simulation of the interaction between PS and HSA was conducted using the AutoDock software package to predict the binding mode of the ligand on the protein for the two main ligand binding sites, I and II; and to confirm the results of the ligand displacement experiments described above. Multiple crystal structures of HSA, i.e., 1BM0, 2BXD and 2BXF were analyzed to ensure the robustness of the simulation method. The crystal structures of 2BXD and 2BXF were chosen for the study as they were reported as a complex with warfarin and diazepam, respectively [35]. Since HSA is known to bind to warfarin at site I and diazepam at site II [6], [7], docking analysis using these structures could reveal the binding preference of PS to either site I or site II of HSA. The docking simulation of 1BM0–PS was analyzed due to the fact that 1BM0 is the highest resolution HSA crystal structure available [42]. At site I of the 1BM0–PS complex, cluster analysis of 100 docking results revealed a total of 7 multi-member conformation clusters at a root-mean-square deviation tolerance of 2.0 Å as shown in Figure 9A. The cluster with the lowest binding energy was also found to be the highest populated cluster, having more than 70% of the analyzed conformations (72 out of 100 conformations). Therefore, it was the most energetically favorable cluster possessing a mean docking energy of about −31.10 kJ mol^−1^. Using the same approach for site II, 24 distinct conformational clusters were obtained. However, the most populated cluster (22 out of 100 conformations) was not the most energetically favorable (−20.88 kJ mol^−1^) cluster. Hence, PS showed a binding preference for binding site I of HSA and these docking results were in good agreement with the displacement results discussed above. Clustering analysis of the 2BXD–PS and 2BXF–PS complexes (Figures 9B and C) also showed similar results in terms of the lowest binding energy and highly populated clusters with site I, strengthening our conclusion about site I of HSA as the primary binding site of PS.

**Figure 10:**
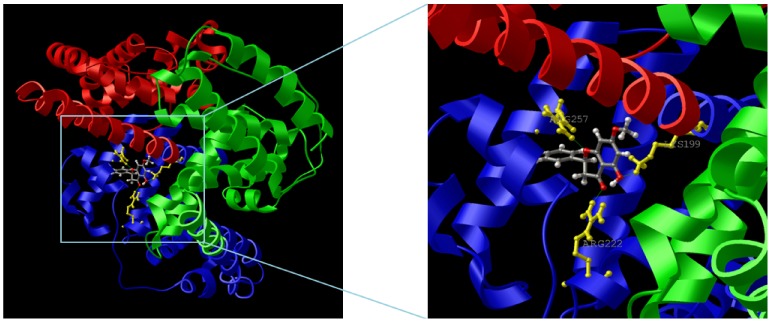
Model of PS docking to site I at subdomain IIA of HSA. Domain structure of HSA (I, red; II, blue and III, green), showing the binding orientation of the lowest docking energy conformation of PS. The zoomed-in view of the binding locus shows the ball-and-stick representation of the ligand and the side-chains of the protein residues (yellow) that form hydrogen bonds (green lines) with PS.

The predicted binding model with the lowest docking energy as obtained from the 1BM0–PS complex (−31.10 kJ mol^−1^) was then used for binding orientation analysis (Figure 10). The binding site (defined as amino acid residues within 5 Å distance with the ligand) was found to be located deep within the protein structure in a hydrophobic cleft walled by the 18 amino acids: Glu-153, Ser-192, Lys-195, Gln-196, Lys-199, Trp-214, Arg-218, Leu-219, Arg-222, Leu-238, Val-241, His-242, Arg-257, Leu-260, Ala-261, Ser-287, Ile-290 and Ala-291. Hydrophobic interactions between hydrophobic residues of the cleft and the benzene rings of the ligand are believed to contribute towards the stability of the docking conformation of PS inside this binding pocket. However, the interaction between PS and HSA cannot be presumed to be exclusively hydrophobic in nature; as there were several polar residues in the proximity of the bound ligand that may participate in polar interactions with the hydrophilic groups of PS. Indeed, three hydrogen bonds were also predicted from the model involving hydrogen atoms of three different amino acid residues of HSA (Lys-199, Arg-222 and Arg-257) and the oxygen atoms of the hydroxyl, carbonyl, and oxacyclohexane groups of PS (Table 3). Hence, it can be concluded that PS binds to a hydrophobic pocket located in subdomain IIA, involving both hydrophobic interactions and hydrogen bonding; in accordance with our thermodynamic data.

**Table 3:** Distance of the predicted hydrogen bonds formed between interacting residues of HSA and PS.

HSA atom	PS atom	Distance, Å
Lys-199: HZ1	O (hydroxyl)	2.182
Arg-222: HH11	O (carbonyl)	2.034
Arg-257: HH22	O (oxacyclohexane)	1.965

## Conclusions

In summary, this study describes a quantitative analysis of PS–HSA interaction. The thermodynamic and molecular modeling data suggested the involvement of van der Waals force, hydrophobic interaction, and hydrogen bonding in the complexation between PS and HSA. Alterations in the protein conformation upon PS binding were evident from multiple spectroscopic results. Binding of PS to HSA increased the thermal stability of the protein and the binding site of PS on HSA was confirmed as site I based on competitive ligand displacement results as well as docking analysis. The biological significance of this work lies in understanding the interaction of PS with HSA, which will be vital for the future designing of PS–derived drugs.
